# Metabolomics unveils the exacerbating role of arachidonic acid metabolism in atherosclerosis

**DOI:** 10.3389/fmolb.2024.1297437

**Published:** 2024-02-07

**Authors:** Sai Ma, Songqing He, Jing Liu, Wei Zhuang, Hanqing Li, Chen Lin, Lijun Wang, Jing Feng, Lei Wang

**Affiliations:** ^1^ Department of Cardiology, Jinling Hospital, Medical School of Nanjing University, Nanjing, China; ^2^ Department of Cardiology, The First School of Clinical Medicine, Southern Medical University, Nanjing, China; ^3^ Department of Emergency Medicine, Jinling Hospital, Medical School of Nanjing University, Nanjing, China; ^4^ Department of Emergency Medicine, The First School of Clinical Medicine, Southern Medical University, Nanjing, China

**Keywords:** atherosclerosis, metabolomics, exacerbating role, metabolism, arachidonic acid

## Abstract

Atherosclerosis is a complex vascular disorder characterized by the deposition of lipids, inflammatory cascades, and plaque formation in arterial walls. A thorough understanding of its causes and progression is necessary to develop effective diagnostic and therapeutic strategies. Recent breakthroughs in metabolomics have provided valuable insights into the molecular mechanisms and genetic factors involved in atherosclerosis, leading to innovative approaches for preventing and treating the disease. In our study, we analyzed clinical serum samples from both atherosclerosis patients and animal models using laser desorption ionization mass spectrometry. By employing methods such as orthogonal partial least-squares discrimination analysis (OPLS-DA), heatmaps, and volcano plots, we can accurately classify atherosclerosis (AUC = 0.892) and identify key molecules associated with the disease. Specifically, we observed elevated levels of arachidonic acid and its metabolite, leukotriene B4, in atherosclerosis. By inhibiting arachidonic acid and monitoring its downstream metabolites, we discovered the crucial role of this metabolic pathway in regulating atherosclerosis. Metabolomic research provides detailed insights into the metabolic networks involved in atherosclerosis development and reveals the close connection between abnormal metabolism and the disease. These studies offer new possibilities for precise diagnosis, treatment, and monitoring of disease progression, as well as evaluating the effectiveness of therapeutic interventions.

## 1 Introduction

Atherosclerosis, a widespread cardiovascular condition, exhibits a multifaceted and heterogeneous pathogenesis (Gao et al., 2016; Khambhati et al., 2018; Sharma et al., 2021). As a powerful investigative tool, Metabolomics has emerged to enhance our understanding and unravel the molecular mechanisms underlying atherosclerosis (Ren et al., 2016; Giunchi et al., 2019; Chen et al., 2021; Yang et al., 2021). Metabolomics encompasses a systematic approach that explores the metabolites within an organism, unravelling intricate associations between metabolites and diseases. This comprehensive methodology provides profound insights into disease development and progression (Zhao et al., 2018; Tian et al., 2022; Xiao et al., 2022; Zhang et al., 2022). First, metabolomics offers comprehensive metabolic profiling by examining compositional and quantitative metabolite changes within cells, tissues, or organisms. This analytical technique unveils metabolic aberrations intimately linked to atherosclerosis, including lipid metabolism, glucose metabolism, amino acid metabolism, and other pivotal pathways crucial for comprehending atherosclerotic pathogenesis. Secondly, metabolomics analysis contributes to the identification and discovery of prospective biomarkers. Early diagnosis of atherosclerosis is of utmost importance, yet conventional clinical examination techniques have limitations. Through metabolomics research, we strive to ascertain metabolic biomarkers associated with atherosclerosis, refining the precision and sensitivity of early diagnosis. This pursuit facilitates early intervention and treatment opportunities for patients.

Matrix-assisted laser desorption/ionization (MALDI) represents an innovative metabolomics analysis approach that surpasses conventional methodologies such as liquid chromatography-mass spectrometry (LC-MS) and nuclear magnetic resonance (NMR) (Schubert and Kostrzewa, 2017; Calvano et al., 2018; Xu et al., 2019; Ashfaq et al., 2022). Initially, MALDI boasts high-throughput capabilities and exceptional sensitivity. Atherosclerosis, being a complex ailment characterized by diverse metabolic pathways and metabolites, necessitates rapid analysis of numerous samples (Ly et al., 2016; Moreno-Gordaliza et al., 2017; Duan et al., 2022). MALDI technology caters to this requirement, enabling high-throughput analysis and fostering a comprehensive understanding of metabolite composition and alterations. Furthermore, MALDI streamlines sample preparation by minimizing the steps involved in sample preprocessing. This reduction mitigates sample loss and variability, bolstering the reliability and repeatability of data. Secondly, MALDI exhibits an advantage in the analysis of small molecule metabolites. Atherosclerosis-related metabolic anomalies primarily involve small molecule metabolites such as lipid metabolites and glucose metabolites. Compared to LC-MS, MALDI excels in ionising and detecting small molecular compounds (Sun et al., 2016; Yan et al., 2017; Torata et al., 2018; Ferey et al., 2019). This attribute empowers the detection of metabolites at lower concentrations, amplifying the detection range and sensitivity of metabolites. In conclusion, MALDI, as a rapid, high-throughput, sensitive, and spatially-resolved metabolomics analysis technique, provides an effective tool for unravelling the molecular intricacies of atherosclerosis. It elucidates the associations between metabolic irregularities and disease progression, ultimately facilitating early diagnosis and treatment of atherosclerosis.

Arachidonic acid and its downstream leukotriene metabolites potentially play pivotal roles in atherosclerosis (Needleman et al., 1986; Piomelli, 1993; Zeldin, 2001). Arachidonic acid, a polyunsaturated fatty acid, generates leukotriene metabolites such as leukotriene B4 (LTB4) and leukotrienes C4, D4, and E4 (LTC4, LTD4, LTE4) through enzymatic catalysis (Bhatt et al., 2017; Gelfand, 2017; Wan et al., 2017). Firstly, these leukotriene metabolites exert substantial regulatory control over inflammatory reactions. Inflammation constitutes a pivotal process in atherosclerosis. The leukotriene metabolites LTC4, LTD4, and LTE4, being potent inflammatory mediators, promote leukocyte adhesion, chemotaxis, and activation, exacerbating the inflammatory response (Gelfand, 2017; Wan et al., 2017). These inflammatory reactions, in turn, stimulate endothelial cell damage and release of inflammatory mediators, establishing a pernicious cycle that accelerates atherosclerosis progression. Secondly, leukotriene metabolites modulate platelet activation and aggregation, a crucial step in thrombus formation within atherosclerosis. The leukotriene metabolite LTB4 stimulates platelet aggregation and releases platelet-activating factors, heightening the risk of thrombus formation (Bhatt et al., 2017; Gelfand, 2017; Wan et al., 2017). Platelet aggregation further intensifies endothelial damage and fosters an augmented inflammatory response, ultimately contributing to atherosclerosis development. Moreover, leukotriene metabolites influence the functionality of smooth muscle cells. Smooth muscle cell proliferation and migration are pivotal features in atherosclerotic lesion development. The leukotriene metabolites LTC4, LTD4, and LTE4 facilitate smooth muscle cell proliferation and migration by binding to receptors on smooth muscle cells, leading to arterial wall thickening and plaque formation.

**FIGURE 1:**
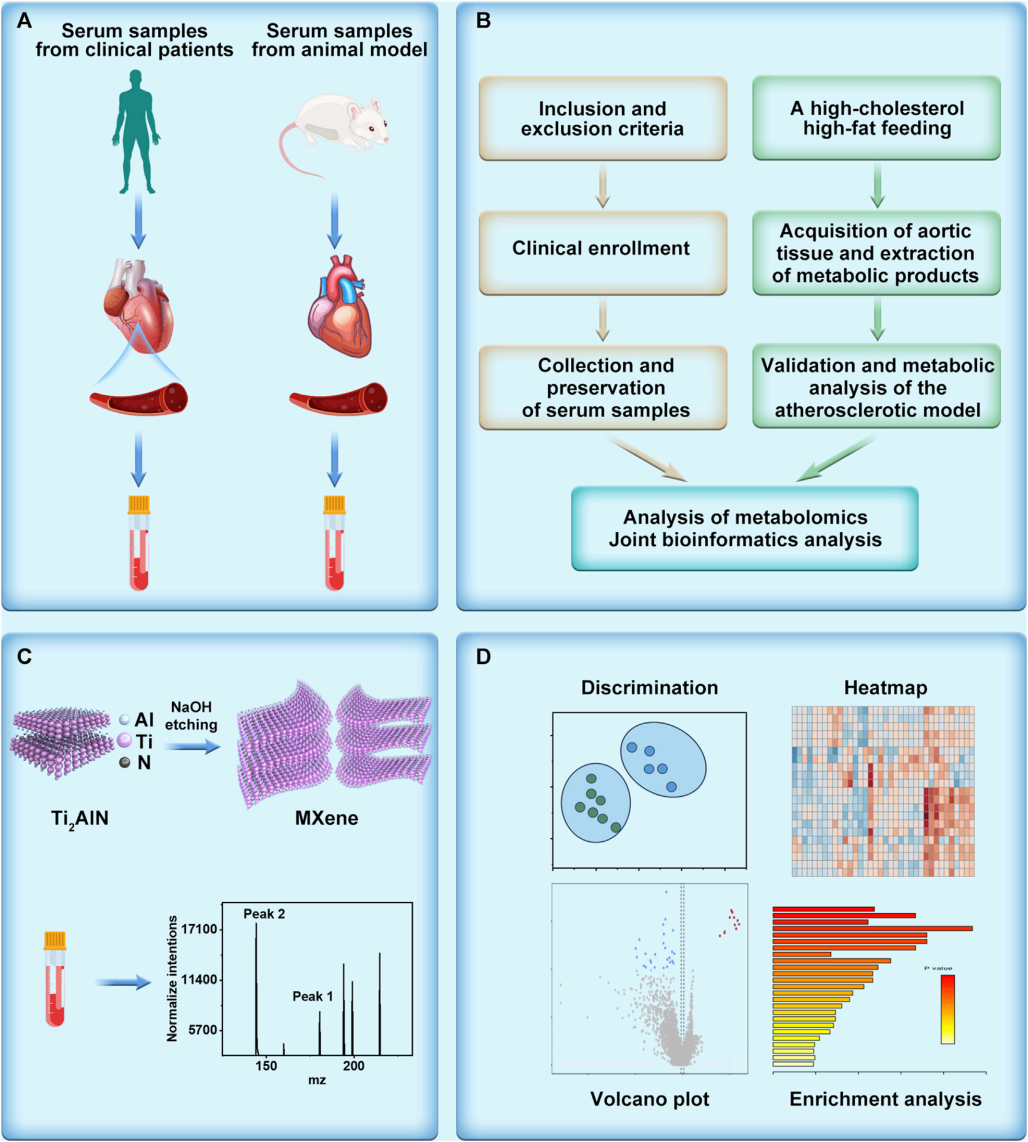
Schematic illustration of metabolomics unveils the exacerbating role of arachidonic acid metabolism in atherosclerosis. **(A)** Collection of serum samples derived from patients with atherosclerosis and serum obtained from an atherosclerosis model constructed using high-fat-fed APOE mice. **(B)** Elucidation of the mechanism of atherosclerosis through metabolomics analysis, depicted in a flowchart. **(C)** Illustration of the synthesis and detection of the metabolomics matrix on atherosclerosis investigation. **(D)** Post-metabolomics analysis, encompassing discrimination, heatmap generation, volcano plot visualization, and enrichment analysis.

Hence, by employing metabolomics analysis utilizing MALDI technology, we conducted comprehensive investigations on clinical samples obtained from atherosclerosis patients and an animal model we constructed (Figures 1A,B). To uncover and analyze the metabolic alterations associated with atherosclerosis, we utilized the exceptional ionization and resolution capabilities of two-dimensional MXene materials. This advanced material, characterized by its unique two-dimensional structure, facilitated the efficient detection of small molecules involved in the metabolic processes of atherosclerosis (Figure 1C). This methodology enabled the identification of distinctive key metabolic pathways implicated in atherosclerosis. Employing strategies like volcano plots and heatmaps, we discerned potential metabolic molecules (Figure 1D). Collaborative screening analysis highlighted the critical involvement of the arachidonic acid pathway. Expanding upon the metabolomics findings, we quantitatively assessed the atherosclerotic effect of arachidonic acid by analyzing arachidonic acid inhibitors and downstream metabolites. Our aspiration is that this study provides meaningful insights into the diagnosis, treatment, and intervention of clinical atherosclerosis. Additionally, we believe that the integration of metabolomics with biological validation affords novel perspectives for researching various diseases, including cardiovascular ailments.

## 2 Materials and methods

### 2.1 Synthesis and characterization of Ti_2_AlN

The fabrication of Ti2AlN, a two-dimensional layered material, was accomplished through a sodium hydroxide etching process. This involved the reaction between 1 g of Ti2AlN material and 10 mL of 3M sodium hydroxide at a temperature of 40°C and a rotational speed of 500 RPM for 12 h. Subsequently, the synthesized material underwent three successive rinses with deionized water. The resulting two-dimensional material was then carefully collected and stored at a temperature of 4°C in a refrigerator for preservation. To examine its microstructure, scanning electron microscopy analysis was performed using a Hitachi SU8600 instrument, operating at a voltage of 10 kV, enabling detailed characterisation of the material’s features and properties.

### 2.2 Construction of AS animal model

A meticulously designed AS animal model was established, comprising 32 ApoE knock-out mice, evenly divided into control and experimental groups. ApoE knockout mice were selected for their altered lipid metabolism genes, rendering them more susceptible to atherosclerosis, thus facilitating a simplified and time-efficient modelling process, albeit at increased expenses. The control group received no intervention, while the experimental group was subjected to a 12-week high-fat, high-cholesterol diet (SY108C) containing 20% fat and 1.25% cholesterol. After the intervention period, samples were meticulously collected from both groups, including blood and aortic tissues. For blood sampling, 2% isoflurane anaesthesia (at a flow rate of 10 mL/min, specific to the anaesthesia equipment employed) was administered, followed by the extraction of blood. The blood was allowed to clot at room temperature for 1–2 h, after which it was centrifuged at 2,500 rpm for 10 min to obtain the serum fraction, which was then stored at −80°C for subsequent analysis. Aortic tissue collection involved carefully positioning the mice in a supine position on a dissecting table, followed by the excision of aortic tissues and their fixation in 10% paraformaldehyde solution for subsequent characterization and assessment.

### 2.3 Masson’s staining, oil Red staining, sirius Red staining

Masson’s staining is a histological technique employed to assess the integrity and pathological alterations of blood vessel walls. It encompasses fundamental stages, including tissue fixation (using 10% buffered formalin), tissue processing (involving dehydration and clearing), tissue embedding (using molten paraffin), tissue sectioning (at a thickness of 4–6 μm), and staining. The specific staining process encompasses deparaffinization (utilizing a xylene solution), dehydration (employing a descending series of ethanol concentrations, such as 100%, 95%, and 80% ethanol), staining (with Masson’s staining solution encompassing acidic fuchsin, aniline blue, and orange G dyes), rinsing (using buffer solution to eliminate excess dye), dehydration (employing an ascending series of ethanol concentrations), clearing (xylene), and mounting (Canada balsam).

The steps involved in Oil Red staining are as follows: Initially, tissue sections are fixed in formaldehyde and subsequently subjected to dehydration, sequentially immersing the sections in an ethanol gradient (70%, 80%, 95%, 100%) for 10 min each, followed by transfer into a clearing agent (xylene) for clearing. Subsequently, the sections are immersed in an oil-red solution, maintained at a temperature of 60–70°C, for 4–8 h to facilitate staining. After staining, the sections are dehydrated in the clearing agent, covered with a coverslip, and mounted.

The procedure for Sirius Red staining involves the fixation of tissue sections in paraformaldehyde, followed by dehydration. The sections are then immersed in an ethanol gradient (70%, 80%, 95%, 100%) for 10 min each and subsequently transferred to a clearing agent (such as xylene) for clearing. Next, the sections are subjected to staining with Sirius Red solution, followed by rinsing with distilled water to eliminate excess dye. The sections are further dehydrated using an increasing series of ethanol concentrations, cleared using a clearing agent, and ultimately mounted utilizing a mounting medium.

### 2.4 Q-PCR and western blotting

The procedure of Western blotting involves several intricate steps. Firstly, the samples undergo separation by SDS-PAGE electrophoresis to resolve the proteins based on their molecular weight. Subsequently, the proteins are transferred onto a polyvinylidene fluoride (PVDF) membrane, facilitating their immobilization for further analysis. Activation of the membrane is achieved through wetting with a 20% methanol solution, ensuring optimal binding efficiency. Following this, specific antibodies are applied to the membrane, facilitating the recognition and binding of the target protein of interest. To enable detection, a secondary antibody labelled with an enzyme, such as horseradish peroxidase (HRP), that exhibits affinity towards the primary antibody is employed. Finally, protein detection is accomplished utilizing sophisticated techniques, including chemiluminescence or substrate staining, which generate a measurable signal indicative of the presence and quantity of the target protein.

The q-PCR procedure entails a series of meticulous steps. Initially, RNA molecules are converted into complementary DNA (cDNA) through reverse transcription, employing the enzyme reverse transcriptase. Subsequently, specific primers and fluorescent probes designed to anneal to the target gene of interest are employed during PCR amplification. The amplification process allows for the exponential replication of the DNA region of interest. Detection of the amplified product is achieved by monitoring the fluorescence signals emitted by the fluorescent probes during PCR cycling. These signals provide a quantifiable measure of the relative expression levels of the target gene in the samples. Ultimately, the gene expression levels are determined by employing the standard curve method, which relates the fluorescence signals to the known concentrations of a reference sample.

### 2.5 Metabolomics analysis

For MALDI-TOF Analysis, initially, 1 µL of the small molecule standard solutions or serum samples was meticulously pipetted onto a stainless steel target plate. Following air-drying at room temperature, 0.5 µL of matrix solution was added to cover the previous spot. After another round of air-drying at room temperature, the samples underwent analysis using an Ultraflex MALDI-TOF MS (Bruker Daltonics, Billerica, MA) operating in a linear positive mode with a laser intensity set at 50%. The molecular weights of the samples, ranging from 80 to 1,000, were meticulously recorded during the analysis. Metabolomics analysis involves various analytical methods such as Principal Component Analysis (PCA), Orthogonal Partial Least Squares Discriminant Analysis (OPLS-DA), Heatmap, Volcano Plot, and Pathway Enrichment. These methods are primarily used to analyze high-throughput mass spectrometry data from both the atherosclerosis group and the control group.

### 2.6 Harvesting of clinical sample collection

Inclusion Criteria: 1) Serum samples must be obtained from patients definitively diagnosed with atherosclerosis to ensure consistency in the disease status of the study subjects: 2) Age range restrictions are applied to maintain sample uniformity while considering the characteristics of atherosclerosis across different age groups; 3) Samples are exclusively selected from patients in a stable period before treatment, minimizing the potential impact of treatment on serum biomarkers and enhancing result reliability. Exclusion Criteria: 1) Patients with other cardiovascular diseases are excluded to ensure the specificity and independence of the study results from atherosclerosis; 2) Individuals with immune system abnormalities are rejected to reduce sample variability caused by immune interference; 3) Patients with severe liver or kidney dysfunction are excluded to mitigate the impact of these factors on serum biomarkers, ensuring result accuracy (Akboga et al., 2015; Hua et al., 2023). The methodology for serum acquisition and preservation is delineated as follows: Haematological samples are meticulously obtained and deposited within receptacles devoid of anticoagulant agents. Subsequently, these samples undergo centrifugation at a velocity of 3,000 rotations per minute for 10 min, thereby resulting in the formation of a supernatant fraction that represents the coveted serum component. Lastly, the serum is diligently safeguarded within a freezer maintained at an ultra-low temperature of −80°C. It is of paramount significance to acknowledge that the collection of clinical samples (2022-RAL-35) has been granted official endorsement by the ethics committee affiliated with the esteemed Medical School of Nanjing University.

### 2.7 Validation of the role of arachidonic acid in atherosclerosis

Aspirin is an irreversible inhibitor of cyclooxygenase (COX), which inhibits the metabolism of arachidonic acid. This inhibition leads to a reduction in the synthesis of prostaglandins (PGs), resulting in antipyretic, analgesic, and anti-inflammatory effects [29,30]. In the experimental group, the atherosclerotic mice received daily injections of aspirin at a dosage of 40 mg/kg for 1 week. The control group, on the other hand, received injections of an equivalent volume of normal saline. After 1 week, the mice from both groups were assessed using Sirius staining.

## 3 Results and discussion

### 3.1 Metabolic fingerprinting of AS using clinical biosamples

To achieve heightened sensitivity for low-abundance metabolites in atherosclerosis samples, we synthesized a two-dimensional MXene (Ti_2_AlN) matrix, harnessing its unique advantages arising from its distinctive two-dimensional structure. This innovative matrix enabled the effective detection of low-abundance metabolites in atherosclerosis samples, as visually depicted in Supplementary Figure S1. Scanning electron microscopy vividly characterized its layered structure, affirming its unique morphology. The elemental composition of the matrix, encompassing Ti, Al, Cl, N, and O, is elucidated in Supplementary Figure S2, while the elemental distribution is apparent in Supplementary Figure S3. Furthermore, we conducted a comprehensive analysis and statistical evaluation of the elemental ratios, as presented in Supplementary Table S1. The aforementioned meticulous morphology and elemental analysis effectively confirmed the integrity and structure of the two-dimensional material, forming a robust basis for subsequent metabolomics analysis in atherosclerosis.

**FIGURE 2:**
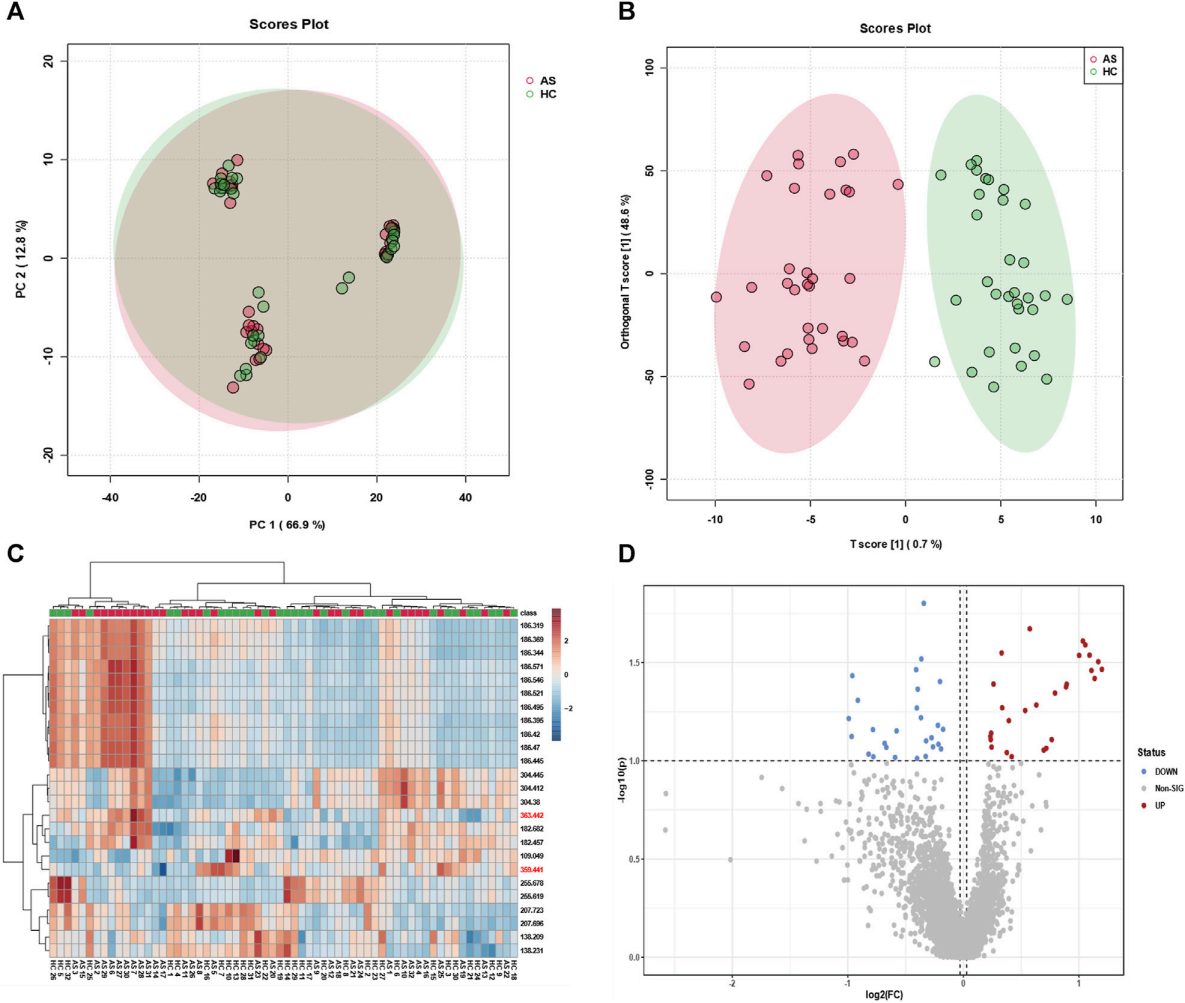
Classification efficacy of clinical samples. Utilizing **(A)** principal component analysis (PCA) and **(B)** orthogonal projections to latent structures discriminant analysis (OPLS-DA), metabolic profiling was performed on serum samples from 38 individuals with atherosclerosis and 35 healthy subjects, yielding classification plots. **(C)** The identified feature mz molecules were utilized to distinguish serum samples from the aforementioned groups, differentiating between the 38 individuals with atherosclerosis and the 35 healthy subjects. **(D)** The volcano plot exhibits upregulated and downregulated factors for serum samples from the 38 individuals with atherosclerosis and the 35 healthy subjects.

To effectively screen potential biomarkers and aberrant metabolic pathways in atherosclerosis, we recruited a cohort comprising 38 atherosclerosis patients and 35 healthy individuals, from whom serum samples were collected to establish distinct groups for metabolomic profiling. Principal Component Analysis (PCA) and Orthogonal Partial Least Squares Discriminant Analysis (OPLS-DA) were employed for data analysis. While PCA focused on reducing data dimensionality and capturing overall variance, OPLS-DA proved more adept at classification and identifying discriminant features distinguishing between the groups. As demonstrated in Figures 2A,B, PCA alone failed to achieve efficient discrimination between atherosclerosis and control groups; however, OPLS-DA successfully distinguished the two groups and revealed significant metabolic disparities between them. Furthermore, a heatmap analysis was performed to unveil key biomolecules implicated in the differentiation of atherosclerosis from the control group. Heatmaps, an invaluable tool in metabolomics, visually depict differences and similarities between various features, enabling rapid identification of critical metabolic pathways or biomarkers. They effectively visualize large-scale metabolic data, provide comprehensive information, and aid in identifying potential associations and trends. Additionally, they facilitate data mining and pattern recognition, expediting researchers’ identification of significant metabolic features relevant to diseases or biological processes. As depicted in Figure 2C, the heatmap revealed distinct metabolic profiles between the serum samples of the 38 athero-sclerosis patients and 35 healthy individuals.

Subsequently, a volcano plot analysis was conducted to further scrutinize the serum samples of the 38 atherosclerosis patients and 35 healthy individuals. Volcano plots, widely employed in metabolomics, visually portray the expression differences and significance of metabolites, facilitating the identification of noteworthy metabolic changes associated with diseases or treatments. Moreover, volcano plots encompass both upregulated and downregulated metabolites, providing comprehensive information and aiding in the discovery of potential biomarkers. As depicted in Figure 2D, the volcano plot unveiled key upregulated and downregulated metabolites in the serum samples of atherosclerosis patients and healthy individuals. These findings strongly indicate significant metabolic disparities between the serum samples of the 38 atherosclerosis patients and 35 healthy individuals. Through the visualization of heatmaps and volcano plots, we successfully demonstrated the differential expression of potential biomarkers between atherosclerosis and control groups, as well as analyzed the trends of upregulation and downregulation. The aforementioned outcomes have laid a solid foundation for subsequent research endeavours. It is crucial to emphasize that our sample size of 38 vs 35 in metabolomic analysis is positioned at an intermediate level (Chinello et al., 2010; Kriegsmann et al., 2015; Laguna et al., 2021; Han et al., 2022), yet remains sufficient for decoding the metabolic fingerprint features of atherosclerosis. Furthermore, our clinical samples (38 vs 35) were meticulously collected following strict admission and exclusion criteria (Experimental section). The MALDI technique, leveraging its high-sensitivity laser desorption ionization capabilities, effectively identifies substantial differences in mz features between the two groups (approximately 30–40 samples ineach group), facilitating the screening of potential biomarkers.

### 3.2 Construction and validation of the atherosclerosis model

**FIGURE 3:**
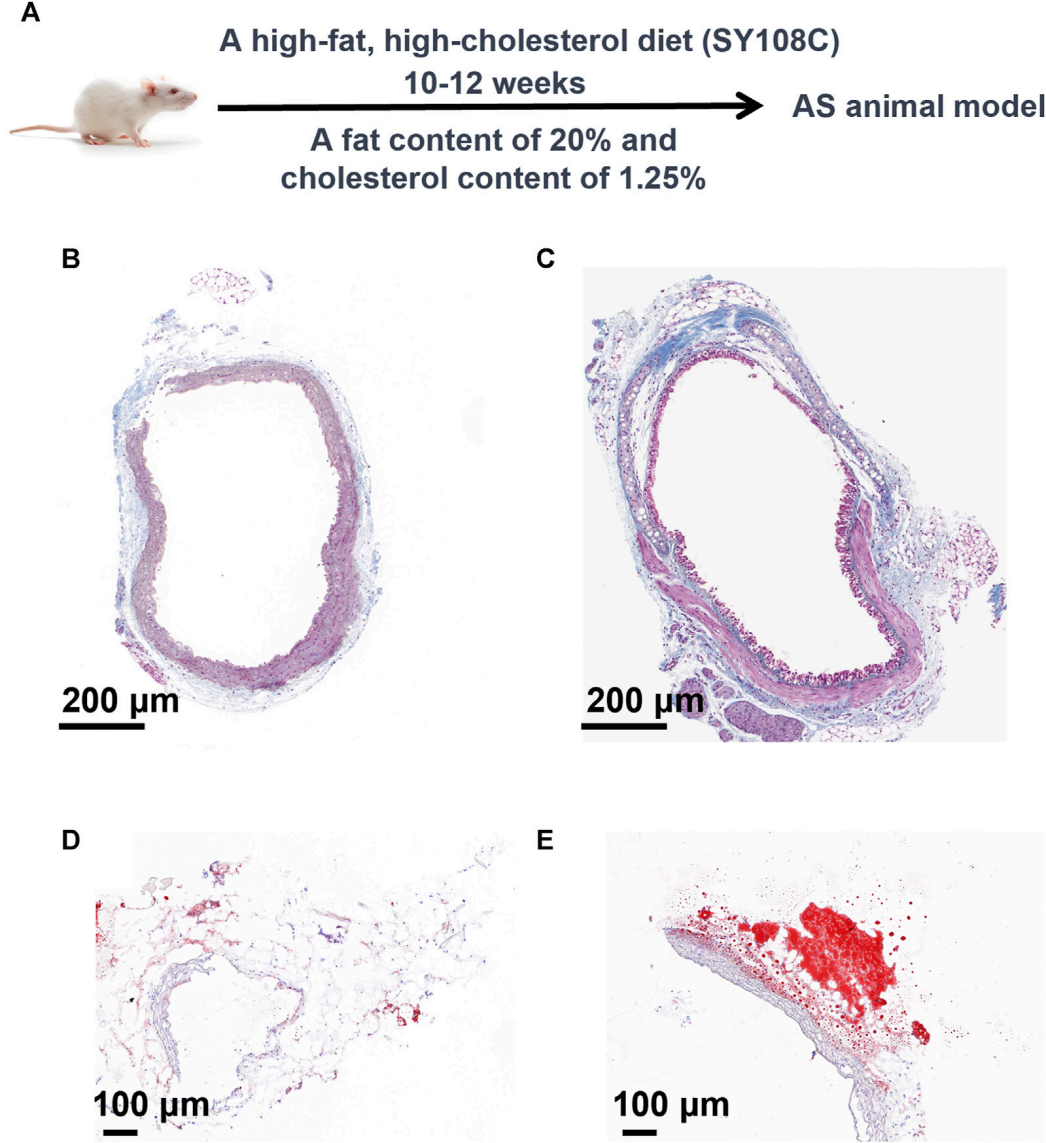
Construction and validation of the atherosclerosis model. **(A)** Methodology and Procedures for Establishing Atherosclerosis. **(B)** Masson’s Staining of the Control Group and **(C)** Masson’s Staining of the Atherosclerosis Group. Analysis of Oil Red O staining **(D)** in the control group and **(E)** in the atherosclerosis group.

To conduct a more precise analysis of potential biomarkers in the metabolomic profile of atherosclerosis and explore disrupted metabolic pathways, we established an atherosclerosis animal model concurrently with a high-fat diet approach. As shown in Figure 3A, we deliberately chose not to use commonly available rodents such as SD rats, Wistar rats, C57 mice, or ICR mice, which possess normal genetic backgrounds, wider availability, lower costs, and less stringent dietary requirements. However, their cholesterol absorption and utilization rates are low, and they exhibit robust plasma cholesterol metabolism capabilities. Consequently, inducing atherosclerosis naturally, even with short-term (within 3 months) utilization of high-fat and high-cholesterol diets, poses a challenge. Instead, we selected ApoE mice, genetically modified to regulate their lipid metabolism and reduce their relative resistance to atherosclerosis. These genetically engineered mice simplify the modelling of atherosclerosis and significantly shorten the modelling time, albeit at a higher cost. The critical second step involved employing an appropriate high-fat feeding method. As depicted in Figure 3A, we adopted a strategy of administering a high-fat and high-cholesterol diet (SY108C), containing 20% fat and 1.25% cholesterol content, for 10–12 weeks to establish a stable atherosclerosis model. To validate the modelling effectiveness of atherosclerosis, we performed Masson’s staining and Oil Red O staining. Masson’s staining evaluates the integrity and lesions of the arterial wall by staining elastic fibers and collagen fibers, while Oil Red O staining is a lipid staining technique that highlights lipid deposition in the arterial wall, particularly lipid plaques in atherosclerotic lesions. These staining techniques provide qualitative and quantitative pathological evaluations, aiding in the understanding of the extent and characteristics of atherosclerosis lesions, thereby offering crucial insights for related research and clinical diagnosis. As depicted in Figures 3B,C, noticeable differences between the atherosclerosis model and the control group are evident. Furthermore, as shown in Figures 3D,E, the lipid content significantly increased in the atherosclerosis group following high-fat feeding. These staining results convincingly demonstrate the success of the atherosclerosis modelling process.

**FIGURE 4:**
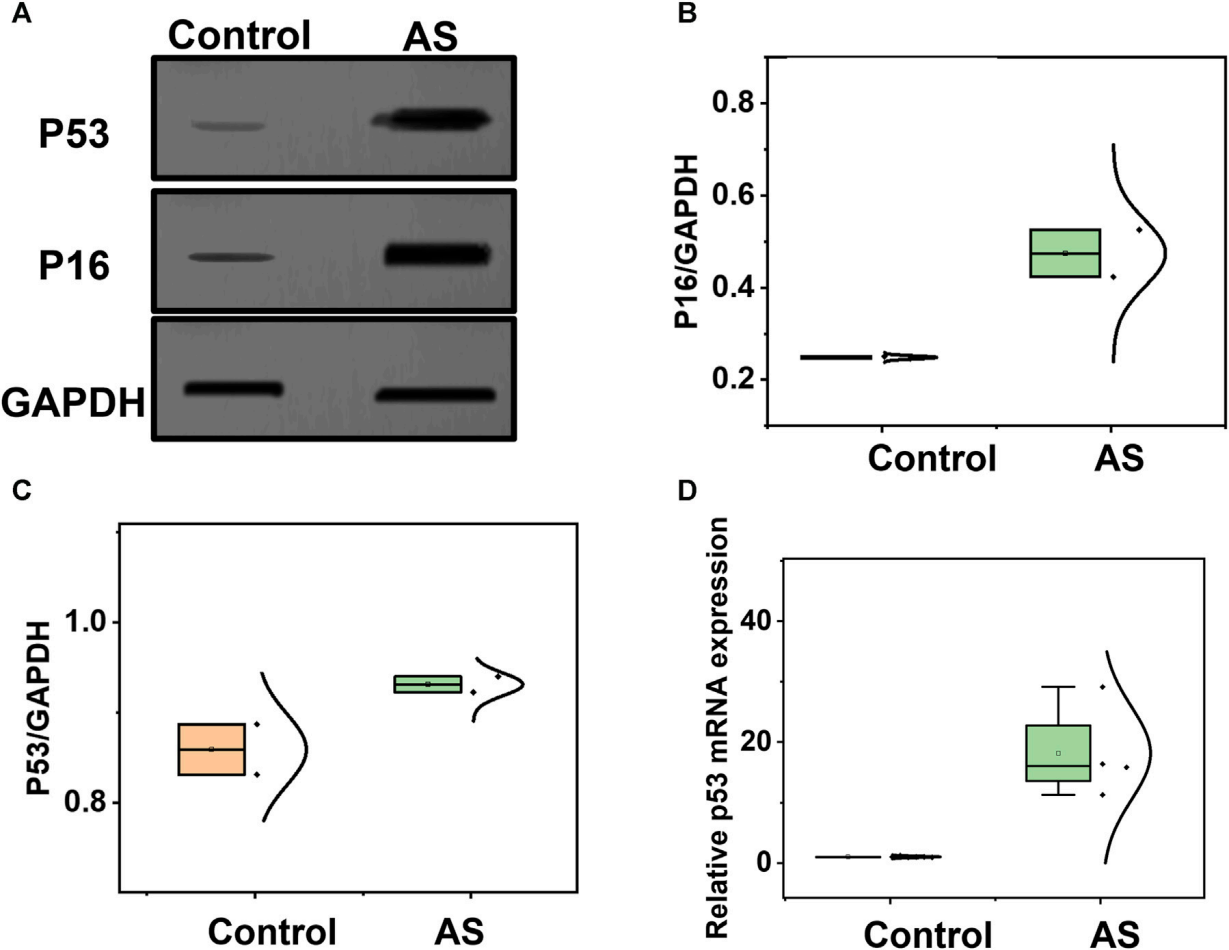
Analysis of potential biomarkers before and after the development of atherosclerosis. **(A)** Western blotting analysis of P16 and P53 in samples from the atherosclerosis model and control group. **(B)** Comparison of P16, and **(C)** P53 between the atherosclerosis group and the control group. **(D)** Comparison of P53 mRNA expression levels between the atherosclerosis group and the control group.

Considering the association between P16, P53, and atherosclerosis, we employed Western blotting to analyze the expression of P16 and P53, as depicted in Figure 4A, using GAPDH as a reference protein. Glyceraldehyde-3-phosphate dehydrogenase (GAPDH), a crucial enzyme in cellular metabolic processes, exhibits stable expression levels in most cells and tissues. By detecting the expression of GAPDH as an internal reference protein, we were able to normalize protein loads in different samples, facilitating the comparison and analysis of the expression levels of the target proteins. We quantified the expression levels of P16 and P53 in atherosclerosis and control groups, as shown in Figures 4B,C, indicating a significant increase in P16 and P53 during the process of atherosclerosis. Additionally, we assessed the mRNA expression levels of P16 and P53 using q-PCR technology. Based on the principles of polymerase chain reaction (PCR), fluorescently labelled probes were utilized to determine the concentration of P16 and P53 mRNA in question. The concentrations of P16 and P53 mRNA were significantly higher in atherosclerosis compared to the control group (Figure 4D; Supplementary Figure S4). The aforementioned staining, Western blotting, and q-PCR characterizations confirm the successful modelling of atherosclerosis, laying the foundation for further metabolomics analysis in the atherosclerosis animal model.

### 3.3 Metabolic fingerprinting of AS using AS animal model

**FIGURE 5:**
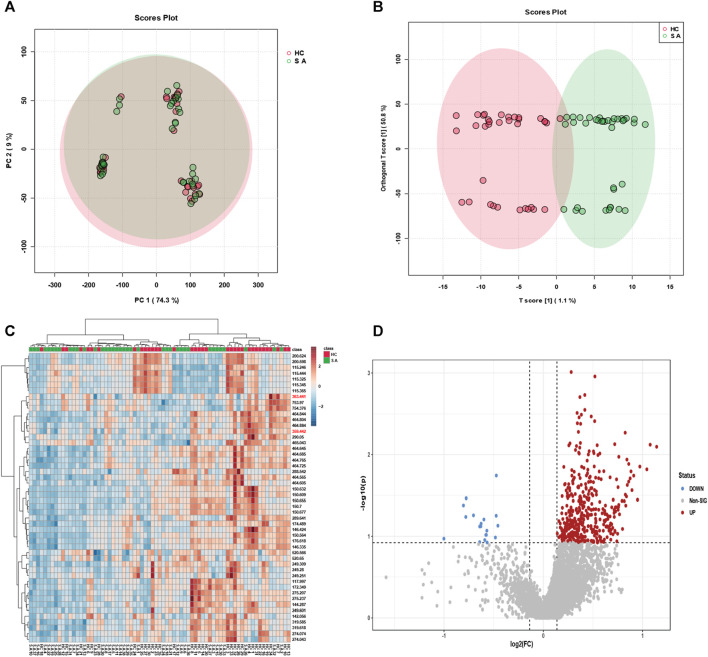
Classification efficacy of serum samples from animal modelling of atherosclerosis compared to the control group. **(A)** Metabolic profiling of serum samples from 32 individuals with modelled atherosclerosis and 32 control subjects using principal component analysis (PCA), and **(B)** orthogonal projections to latent structures discriminant analysis (OPLS-DA), resulting in classification plots. **(C)** The identified feature mz molecules were utilized to discriminate between serum samples from the 38 individuals with atherosclerosis and 35 healthy subjects. **(D)** The volcano plot illustrates upregulated and downregulated factors in serum samples from the 32 individuals with modelled atherosclerosis and 32 control subjects.

Expanding upon the atherosclerosis animal model, to comprehensively screen potential biomarkers and disrupted metabolic pathways associated with atherosclerosis, a collection of 32 serum samples from atherosclerosis animal models and 32 control group samples were acquired. Principal Component Analysis (PCA) and Orthogonal Partial Least Squares Discriminant Analysis (OPLS-DA) methods were employed for classification. As depicted in Figures 5A,B, PCA failed to effectively distinguish between atherosclerosis and control groups, whereas the OPLS-DA method achieved significant separation between the two groups, indicating distinctive metabolic profiles between atherosclerosis and control groups, consistently aligning with observed trends in clinical samples. Moreover, a heatmap was utilized to visualize key metabolites that differentiate the 32 atherosclerosis animal model samples from the 32 control group samples, as demonstrated in Figure 5C, prominently illustrating the intergroup differences in various metabolites, thereby facilitating subsequent metabolic analyses. Additionally, a volcano plot was employed to analyse upregulated and downregulated markers in the serum samples of the 32 atherosclerosis animal models compared to the 32 control group samples, as depicted in Figure 5D. The analyses conducted on the 32 atherosclerosis animal model samples and 32 control group samples, including heatmap and volcano plot analyses, in conjunction with clinical metabolomics analysis, aim to identify potential biomarkers and disrupted metabolic pathways associated with atherosclerosis. Due to metabolic heterogeneity, there may be significant metabolic differences among patients. The advantage of animal models lies in the controllable experimental conditions, including factors such as diet, exercise, and other lifestyle variables. This controlled experimental environment helps minimize external factors’ interference with metabolic analysis, ensuring more reliable results. Combining animal models with patient serum samples can provide a more extensive and comprehensive metabolic profile, which contributes to uncovering the pathogenesis of atherosclerosis. This comprehensive approach will significantly contribute to unravelling the pathogenesis of metabolomics and provide valuable insights for the prevention, treatment, and management of atherosclerosis.

### 3.4 Screening of biomarkers and disrupted metabolic pathways associated with atherosclerosis

**FIGURE 6:**
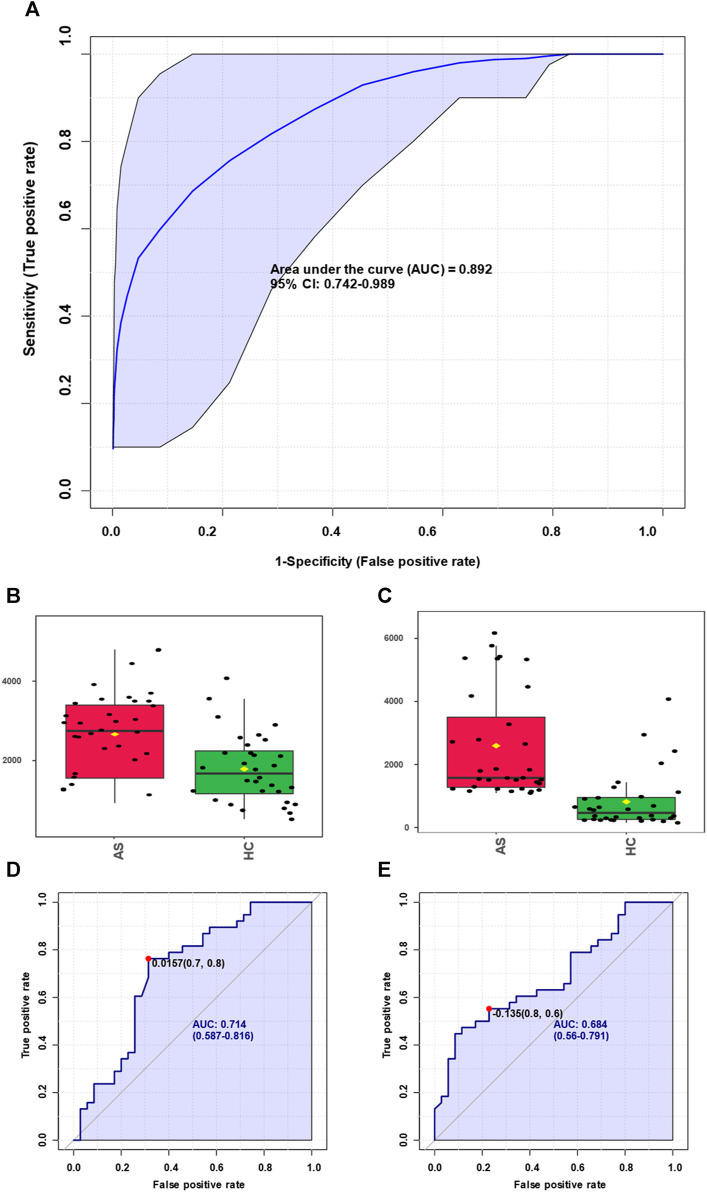
Screening of biomarkers for atherosclerosis and corresponding diagnosis capability. **(A)** Summarizing the selected panel of biomarkers derived from the integration of metabolomics results from clinical samples and animal models, enabling discrimination between atherosclerosis and the control group with an area under the curve (AUC) of 0.892. **(B)** Significant elevation of arachidonic acid molecules in atherosclerosis. **(C)** Arachidonic acid molecules were used for discrimination between atherosclerosis and the control group, yielding an AUC value of 0.714. **(D)** Marked increase of leukotriene B4 molecules in atherosclerosis. **(E)** Leukotriene B4 molecules were used for discrimination between atherosclerosis and the control group, resulting in an AUC value of 0.684.

Expanding upon the integrated metabolomics analysis of clinical samples and animal models of atherosclerosis mentioned earlier, we have identified overlapping potential biomarkers for the diagnosis of atherosclerosis. Moreover, we assessed the diagnostic efficiency using receiver operating characteristic (ROC) curves, which provide a comprehensive evaluation of the sensitivity and specificity of the diagnostic test for atherosclerosis. Sensitivity pertains to the test’s ability to accurately identify patients with atherosclerosis, while specificity relates to its capacity to correctly exclude individuals without the condition. By considering both factors, the ROC curve allows us to assess the performance of the diagnostic test based on the curve’s shape and the area under it, known as the area under the curve (AUC). As illustrated in Figure 6A, the obtained AUC was 0.892, indicative of high diagnostic performance and significant reference value. Furthermore, we observed significant expression of arachidonic acid in both atherosclerosis and control groups, as depicted in Figure 6B. The diagnostic efficiency of arachidonic acid alone was 0.714 (Figure 6C). Similarly, leukotriene B4 (LTB4), as a downstream product of arachidonic acid, exhibited significant expression in both atherosclerosis and control groups, as illustrated in Figure 6D. The diagnostic efficiency of LTB4 alone was 0.684 (Figure 6E). These findings suggest that integrating metabolomics analysis results from clinical samples and animal models enables the effective diagnosis of atherosclerosis. Moreover, the identification of significantly elevated levels of arachidonic acid and its downstream product, LTB4, in atherosclerosis presents opportunities for further exploration of disrupted metabolic pathways in this condition.

**FIGURE 7:**
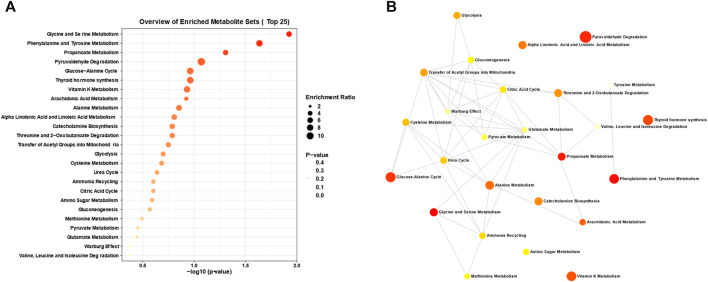
Analysis of disrupted metabolic pathways in atherosclerosis. **(A)** Enrichment analysis and **(B)** interconnections between the dysregulated metabolic pathways closely associated with atherosclerosis, derived from the metabolomics analysis of the aforementioned clinical biosamples and animal models.

In our investigation of atherosclerosis, we have identified arachidonic acid and its downstream product, leukotriene B4, as potential biomarkers. To gain deeper insights, we conducted an enrichment pathway analysis, which aims to identify metabolic pathways that are significantly enriched under specific conditions. This analytical approach allows for a better understanding of the activity and regulatory mechanisms of metabolic pathways in different physiological states or disease processes. Through this exploration, we can unravel the regulatory networks and key nodes of metabolism, thus enhancing our understanding of organismal metabolic function. As illustrated in Figure 7A, we have discovered several enriched metabolic pathways, including arachidonic acid metabolism, glycine and serine metabolism, propanoate metabolism, pyruvaldehyde degradation, the glucose-alanine cycle, thyroid hormone synthesis, and vitamin K metabolism. Notably, arachidonic acid metabolism is closely linked to both arachidonic acid and leukotriene B4. Furthermore, we analyzed the network diagram of metabolic pathways (Figure 7B), which provides a graphical representation integrating metabolic pathways, metabolites, and their interactions. This visual framework aids in comprehending the relationships and interactions between metabolic pathways, as well as the structure and functionality of the overall metabolic network. Through this analysis, we have gained valuable insights into the spatial relationships of various relevant metabolic pathways in atherosclerosis.

### 3.5 Validation of the promoting role of arachidonic acid and its downstream metabolites in atherosclerosis

**FIGURE 8:**
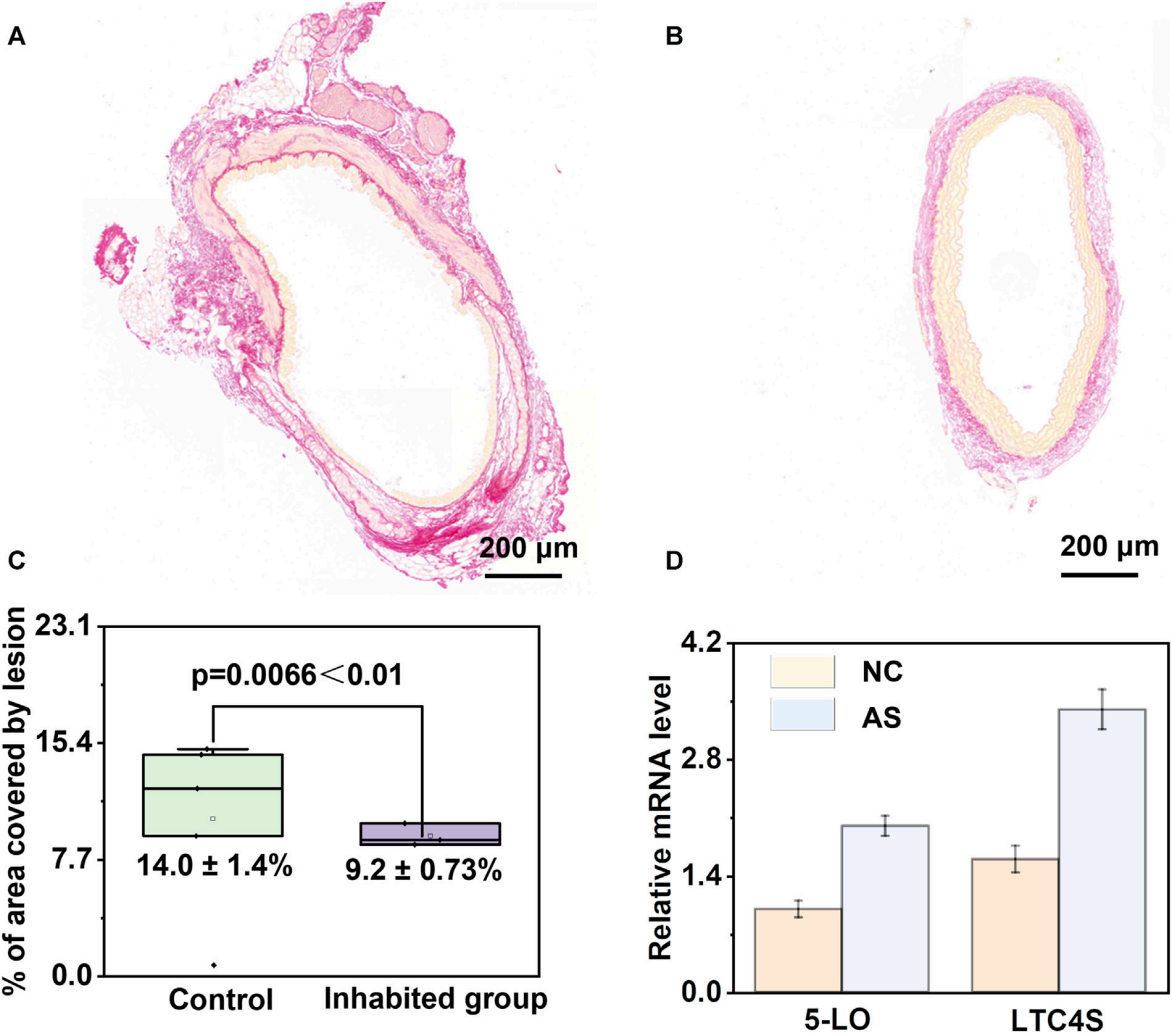
Validation of the pivotal role of arachidonic acid metabolism disruption in atherosclerosis. **(A, B)** Analysis of Sirius-red staining before and after the administration of arachidonic acid inhibitor in atherosclerotic mice. **(C)** Analysis of plaque area before and after inhibitor administration, demonstrating effective mitigation of atherosclerosis upon inhibitor administration. **(D)** Significant upregulation of 5-lipoxygenase (5-LO) and LTC4 synthase (LTC4S) in the atherosclerosis group compared to the control group.

The above results indicate the regulatory role of arachidonic acid and its downstream products in the process of atherosclerosis. Therefore, Sirius staining is also employed for the assessment of collagen content. When observed through a standard optical microscope, collagen fibres within tissues such as the heart and blood vessels exhibit vivid red staining, whereas muscle fibres exhibit distinct yellow staining. The accompanying Figures 8A,B illustrates the results of Sirius staining performed before and after the application of an arachidonic acid inhibitor in atherosclerosis mice (Supplementary Figure S5). In Figure 8C, the collagen area within the image was quantified using the ImageJ software and subsequently divided by the total plaque area to derive the percentage of collagen content within the plaque. A higher relative collagen content within the plaque indicates a greater degree of stability, thereby implying that the introduction of the Arachidonic acid inhibitor reduces plaque stability. Additionally, we quantified the area percentage of plaques before and after applying the arachidonic acid inhibitor and found that the area decreased from 14.0% ± 1.4% to 9.2% ± 0.73%. We assessed the levels of 5-lipoxygenase (5-LO) and LTC4 synthase (LTC4S) concentrations, and intriguingly, observed a substantial upregulation in their abundance within the atherosclerosis group when compared to the control group (Figure 8D). These findings strongly imply a parallel surge in the levels of downstream metabolites derived from arachidonic acid as well. During the development of atherosclerosis, arachidonic acid participates in inflammatory responses and cellular signalling through various pathways, influencing the pathological changes in the arterial wall. Once endothelial cells in the arteries are damaged or stimulated, arachidonic acid can be released and converted into leukotriene B4 (LTB4), a potent inflammatory mediator. LTB4 can attract and activate white blood cells, leading to the occurrence of inflammation. Therefore, we speculate that arachidonic acid and its downstream product, leukotriene B4, may promote the adhesion and migration of white blood cells to the arterial wall, thereby facilitating the formation of inflammatory cell plaques within the blood vessels. Additionally, leukotriene B4 can stimulate the release of platelet-aggregating factors and other inflammatory mediators, further accelerating plaque formation and progression. Therefore, we hypothesize that arachidonic acid and its metabolite leukotriene B4 play important regulatory and mediating roles in the pathological process of atherosclerosis.

## 4 Conclusion

In summary, our innovative metabolomics screening strategy has demonstrated remarkable efficacy in enabling precise diagnosis, identifying biomarkers, and conducting comprehensive analysis of metabolic pathways in atherosclerosis. Our diagnostic methodology, utilizing advanced techniques such as OPLS-DA, heatmaps, and volcano plots, has achieved a high area under the curve (AUC = 0.892) for atherosclerosis classification. This has provided us with visual representations and in-depth scrutiny of the upregulation/downregulation patterns of pivotal molecules involved in the intricate processes of atherosclerotic development. Through meticulous enrichment analysis, we have discovered a significant elevation in arachidonic acid and its downstream metabolite, leukotriene B4, in atherosclerosis. Rigorous biomarker validation has yielded compelling evidence that elucidates the essential regulatory role of the arachidonic acid metabolic pathway in atherosclerosis, utilizing potent arachidonic acid inhibitors and dissecting downstream metabolites. Overall, our metabolomics approach has provided profound insights into the molecular intricacies and underlying genetic foundations of atherosclerosis, paving the way for novel avenues and promising prospects in disease prevention and therapeutic intervention. We anticipate that the widespread dissemination of our research findings will contribute to the heightened precision and efficiency of clinical medicine, offering invaluable guidance and unwavering support for the prevention, treatment, and management of related ailments.

## Data Availability

The original contributions presented in the study are included in the article/Supplementary Material, further inquiries can be directed to the corresponding authors.
