# Supplementary material for: Metabolomics unveils the exacerbating role of arachidonic acid metabolism in atherosclerosis
Source: Front Mol Biosci. 2024 Feb 7;11:1297437. doi: 10.3389/fmolb.2024.1297437 (PMC10879346; doi:10.3389/fmolb.2024.1297437)
Supplement: Supplementary file 1 [file DataSheet1.PDF]

Supporting information

Metabolomics Unveils the Exacerbating Role of Arachidonic Acid Metabolism in Atherosclerosis

Sai Ma[1,2], Songqing He[1,2], Jing Liu[1,2], Wei Zhuang[1,2], Hanqing Li[1,2], Chen Lin[1,2], Lijun Wang[1,2], Jing Feng[3,4*] and Lei Wang[1,2*]

[1]Department of Cardiology, Jinling Hospital, Medical School of Nanjing University, Nanjing, 210002, PR China
[2]Department of Cardiology, the First School of Clinical Medicine, Southern Medical University, Nanjing, 210002, PR China
[3]Department of Emergency Medicine, Jinling Hospital, Medical School of Nanjing University, Nanjing, 210002, PR China
[4]Department of Emergency Medicine, the First School of Clinical Medicine, Southern Medical University, Nanjing, 210002, PR China

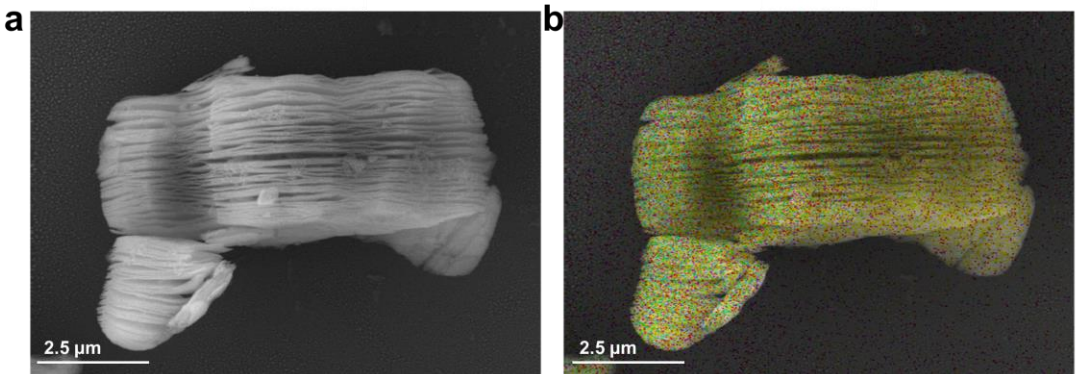

Figure S1. a） SEM photography and b) elemental merged photograph of MXene (Ti_2AlN) matrix used for metabolic of atherosclerosis disorder.

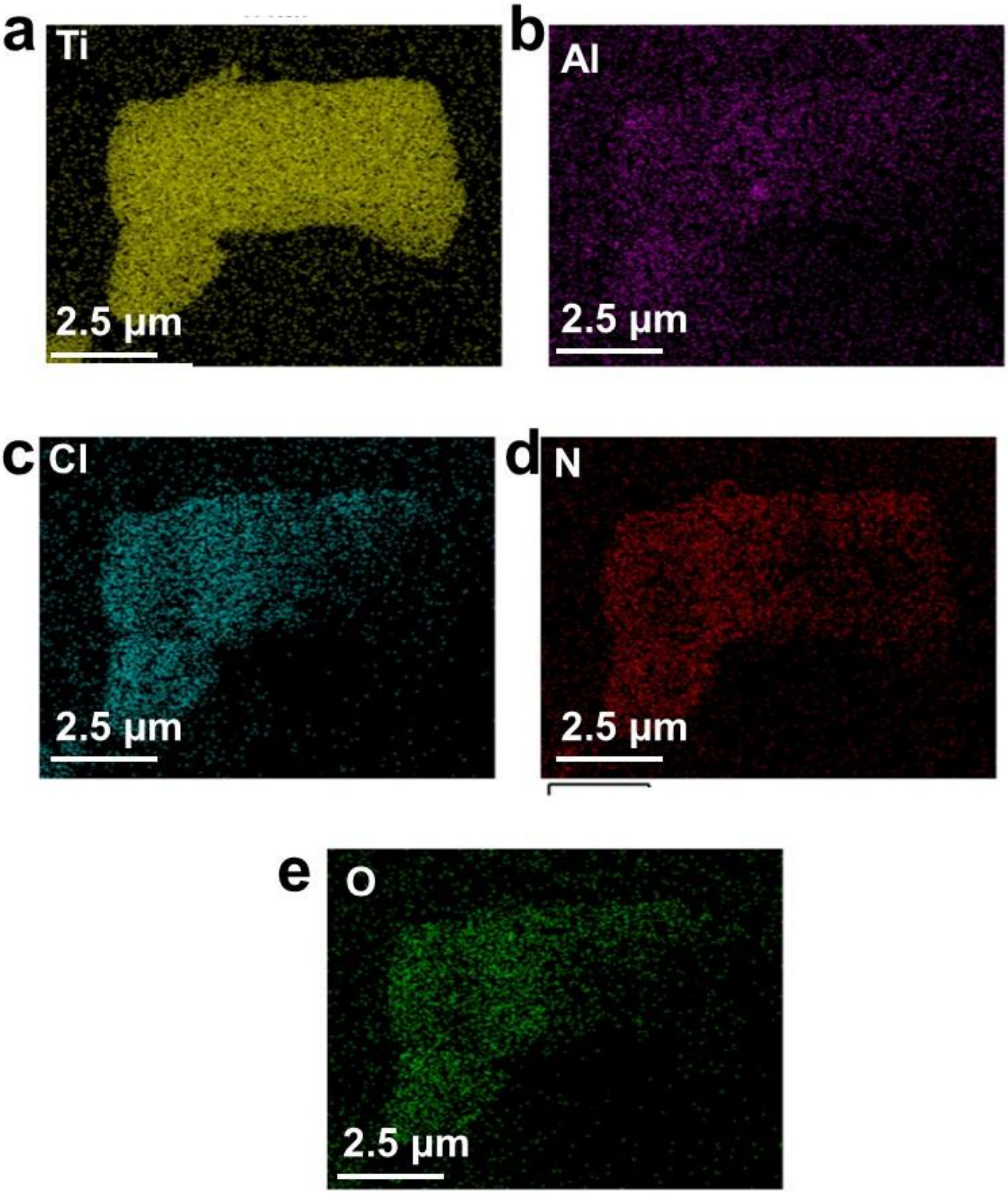

Figure S2. Elemental analysis of MXene (Ti_2AlN) matrix used for metabolic of atherosclerosis disorder, including elements a) Ti, b) Al, c) Cl, d) N and e) O.

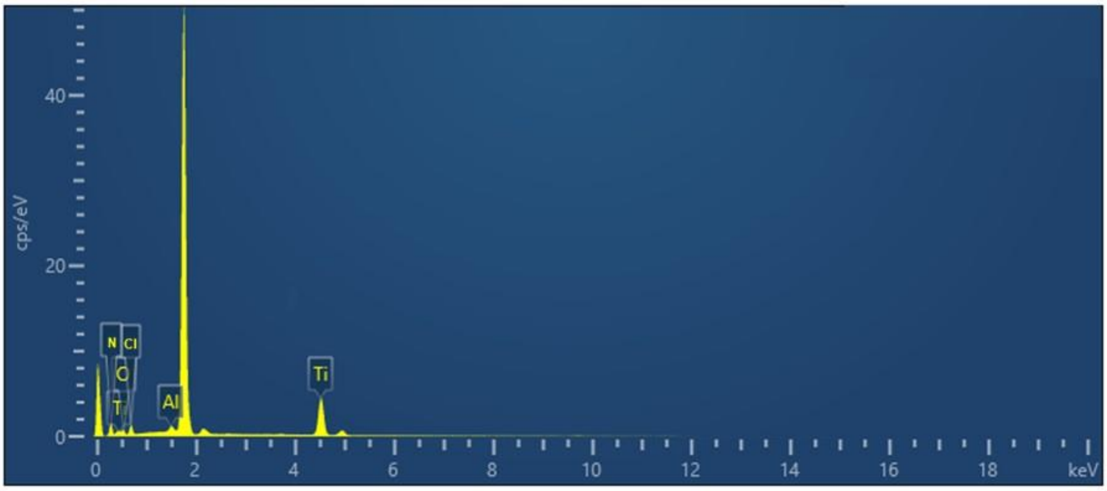

Figure S3. Elemental distribution of MXene (Ti_2AlN) matrix used for metabolic of atherosclerosis disorder, including elements Ti, Al, Cl, N and O.

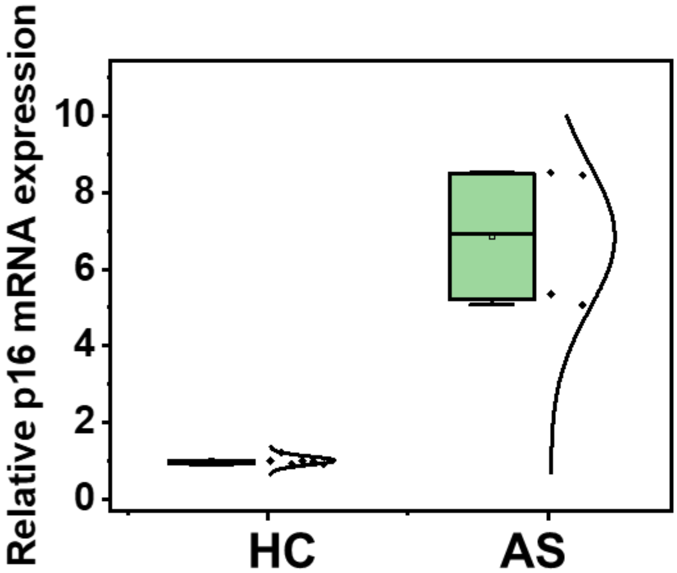

Figure S4. Analysis of mRNA expression levels of P16 in the atherosclerosis group and the control group using Quantitative Polymerase Chain Reaction (Q-PCR) methodology.

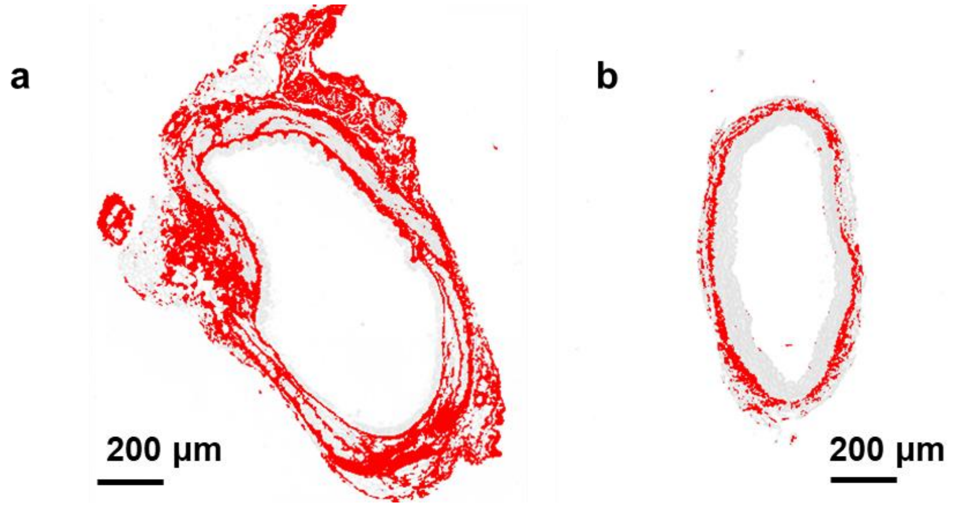

Figure S5. Photographs of atherosclerotic plaques obtained using ImageJ software a) before and b) after the injection of an arachidonic acid inhibitor.

Table 1. Elemental composition analysis of MXene (Ti_2AlN) matrix used for metabolic of atherosclerosis disorder.

**Element**	**Type**	**wt%**	**Wt % Sigma**	**At%**
**N**	K	22.95	0.47	41.78
**O**	K	10.95	0.59	14.97
**Cl**	K	16.60	0.41	19.10
**Al**	K	4.42	0.11	3.58
**Ti**	K	45.08	0.48	20.58
**Total amount**		100.00		100.00
